# Terrain-Height-Blind Quadrupedal Locomotion Through Privileged Teacher–Student Learning and a Shared Gait-Structured Central Pattern Generator

**DOI:** 10.3390/biomimetics11090677

**Published:** 2026-09-19

**Authors:** Rui Qin, Yongbiao Hu, Yaguang Zhu

**Affiliations:** Key Laboratory of Road Construction Technology and Equipment, Ministry of Education, Chang’an University, Xi’an 710064, China; hybiao@chd.edu.cn (Y.H.); zhuyaguang@chd.edu.cn (Y.Z.)

**Keywords:** quadrupedal locomotion, central pattern generator, privileged learning, recurrent policy, proprioception, rough terrain, slope transfer, biomimetics

## Abstract

Terrain-aware training can support locomotion without requiring terrain-height input at deployment. We present a quadrupedal control framework that combines privileged teacher–student learning with a shared gait-structured sensor-consistency central pattern generator (GSC-CPG). A recurrent student uses proprioception, contact, commands, action history, and rhythmic state to produce 12-dimensional modulation of the controller. Teacher and student act through the same phase and foot-target pathways. We evaluated the framework in Go2 simulations with disturbances applied to actor observations and direct simulator feedback retained downstream. Across three matched policy pairs, each with a shared behavior-cloned initialization, continued teacher guidance reduced the pooled rough-terrain failure rate from 1.479 to 1.097 events per robot-minute. Tracking, body tilt, forward speed, and action saturation also improved, while action variation increased. These locomotion benefits extended to a held-out roughness amplitude. On an unseen 5° uphill slope, guided policies had no physical falls over 384 robot-minutes, compared with nine for guidance-off policies. Component tests showed selective, reversible phase responses and a support-related speed–stability trade-off. The results support a shared rhythmic interface for transferring terrain-aware supervision to terrain-height-blind locomotion under the tested simulation conditions.

## 1. Introduction

Locomotion over uneven ground benefits from terrain preview. Local height observations allow a controller to adjust swing clearance, phase, and foot placement before contact, but they also create a runtime dependence on an exteroceptive sensing and terrain-estimation pipeline. Occlusion, reflective surfaces, and inaccurate reconstruction can weaken that pipeline [1]. Without terrain-height input, the same instantaneous body and joint state may precede different ground interactions, so the deployed control problem becomes partially observable. The central question of this study is whether terrain-height information can guide learning without becoming an input to the deployed actor.

Biological locomotion provides a useful organization for this problem. Rhythm-generating activity can persist without rhythmic sensory input, while descending drive, interlimb patterning, sensory feedback, and stance–swing regulation shape the realized movement [2,3,4,5,6,7,8]. Interlimb symmetry further constrains phase relations associated with canonical gaits [9]. This separation between intrinsic rhythm and sensory correction has motivated central pattern generator (CPG) controllers that combine oscillator phase with vestibular and contact feedback on irregular terrain [10,11,12]. Adaptive and self-organized oscillators can entrain timing and coordination [13,14,15], and a related estimation view treats the rhythmic state as an internal prediction corrected by uncertain sensory evidence [16].

Recent learning-based controllers place oscillator variables under descending policy control. Structured interfaces have been used to learn amplitude and frequency modulation, decentralized coordination, gait transitions, and blind locomotion repertoires [17,18,19,20]; adaptive-frequency CPG timing has also been combined with multi-scale feedback and model-based force control [21]. In parallel, dynamics randomization, actuator modeling, and massively parallel simulation have enabled robust learned locomotion [22,23,24,25]. Privileged learning addresses missing runtime information more directly: terrain-aware teachers, rapid adaptation modules, and recurrent actors use recent state–action history to encode properties that are absent from the current observation [26,27,28]. These approaches suggest that terrain can shape a policy during training and later be inferred indirectly from the consequences of robot–ground interaction.

Terrain-height-blind locomotion still relies on information about the robot and its contact with the ground. Body motion can be estimated by combining inertial measurements, leg kinematics, and contact [29,30]. Foot contact can be inferred from dynamics and kinematics, learned from proprioceptive signals, or combined with model-based event logic [31,32,33,34]. Here, terrain-height-blind means that local height samples, elevation maps, depth images, and teacher actions are absent from the deployed actor input. Body and joint state, binary contact, commands, action history, and controller state remain available. The recurrent actor uses their history to respond to the consequences of robot–ground interaction.

We combine a terrain-aware teacher and privileged critic with a recurrent student and a shared gait-structured sensor-consistency CPG (GSC-CPG). The teacher and critic receive 128-D observations, including 60 local height samples; the gated recurrent unit (GRU) student uses the 68-D runtime subset. Teacher and student actors produce the same 12-D amplitude, cadence, and sagittal foot-placement commands and share the downstream rhythmic controller, inverse kinematics, and joint servo. This interface connects terrain-aware supervision to terrain-height-blind execution through the same control variables.

The study makes three contributions.

(1) A teacher–student framework transfers terrain-aware action supervision through a shared rhythmic interface, while keeping terrain-height information confined to the teacher and critic during training.
(2) The GSC-CPG combines common and relative-phase coordinates with bounded sensory corrections and separate sagittal and vertical foot-target pathways. This gives the policy explicit rhythmic control variables while retaining contact-responsive feedback downstream.
(3) Matched student-policy comparisons quantify the benefit of continued teacher guidance after behavior-cloning initialization. Roughness and slope evaluations, a comparison of complete training procedures, and component tests describe the operating range and performance trade-offs of the framework.

## 2. Materials and Methods

### 2.1. Overall Framework and Evaluation Design

**Figure 1:**
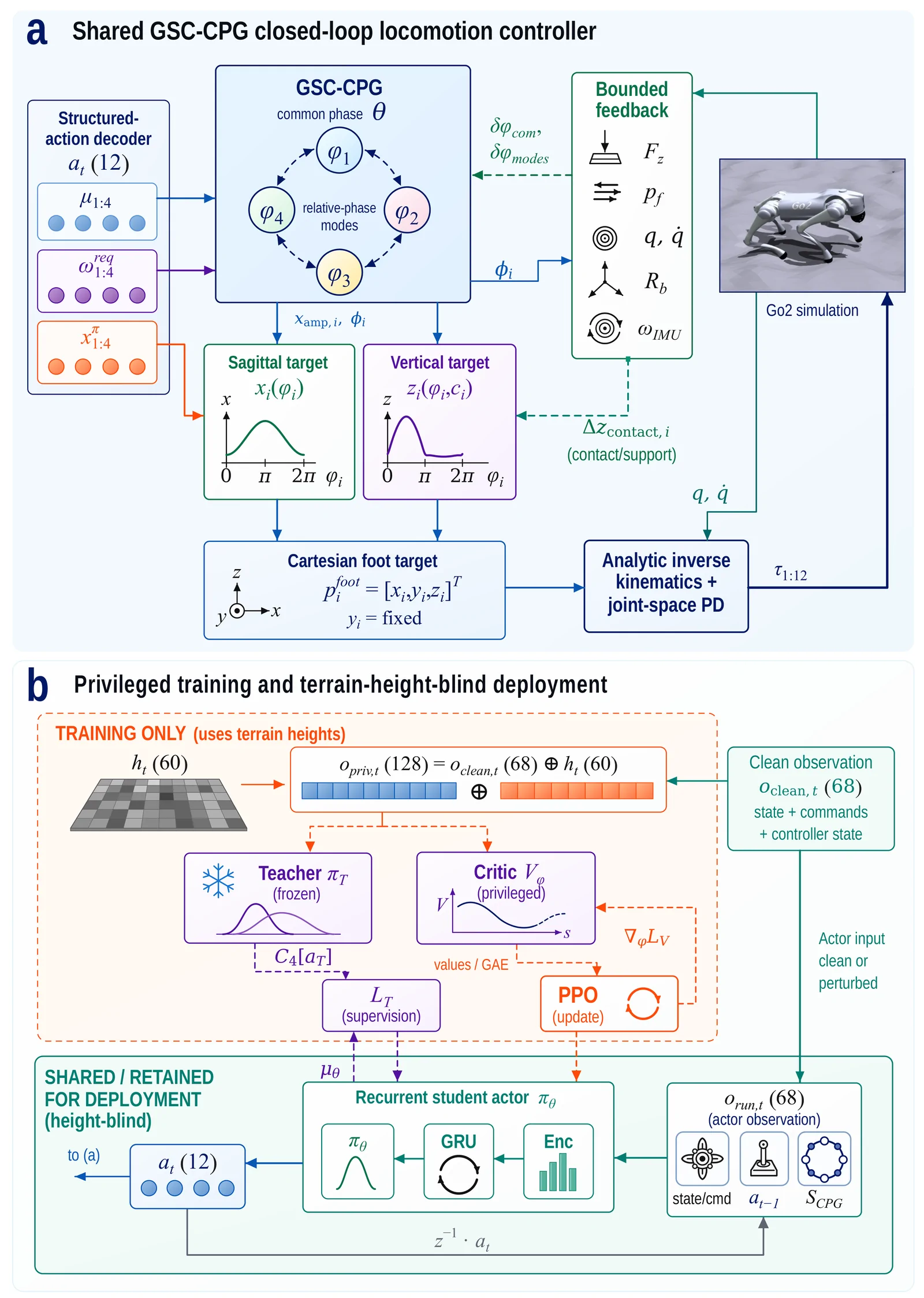
Shared GSC-CPG controller and learning architecture. (**a**) Per-leg amplitude, cadence, and sagittal-placement commands generate foot targets, converted to torques by inverse kinematics and PD control. Inset: Go2 simulation. (**b**) The frozen teacher and trainable critic receive 128-D observations, including 60 terrain heights; the GRU student uses 68-D inputs and learns through teacher supervision and PPO. Deployment retains only the student and controller. *Abbreviations:* GSC-CPG, gait-structured sensor-consistency central pattern generator; GRU, gated recurrent unit; PPO, proximal policy optimization; PD, proportional–derivative; GAE, generalized advantage estimation; IMU, inertial measurement unit; Enc, encoder; cmd, command.

Figure 1 summarizes teacher training, behavior cloning (BC), and online student optimization. At deployment, the recurrent student acts through the same GSC-CPG as its teacher. The main experiment compares students with and without continued guidance from paired BC initializations; further evaluations examine held-out terrain and controller responses.

### 2.2. Shared Gait-Structured Central Pattern Generator

The teacher and student policies in the learning comparisons use the same Full GSC-CPG. The controller configurations varied in the component tests are specified in Section 2.4.

#### 2.2.1. Structured Action and Phase Coordinates

At each 100 Hz policy step, let ${\tilde{a}}_{t}\in R^{12}$ be the actor output and $C_{b}\left(z\right)=\mathrm{clip}\left(z,-b,b\right)$. The environment stores $a_{t}^{100}=C_{100}\left({\tilde{a}}_{t}\right)$, while the controller executes $u_{t}=C_{4}\left(a_{t}^{100}\right)/4$ through three four-leg command channels:

(1) $$D\left(u_{t}\right)={\left[\mu_{t}^{⊤},{\left(\omega_{t}^{\mathrm{req}}\right)}^{⊤},{\left(x_{t}^{\pi }\right)}^{⊤}\right]}^{⊤}\in R^{12}.$$

The stored $a_{t}^{100}$ enters the next observation and action-rate reward. Saturation diagnostics use the pre-clip output and count components with magnitude above the execution limit of 4. The decoder filters cadence and bounds every physical channel as summarized in Table 1; yaw compensation remains a separate bounded term in the foot-target generator.

**Figure 2:**
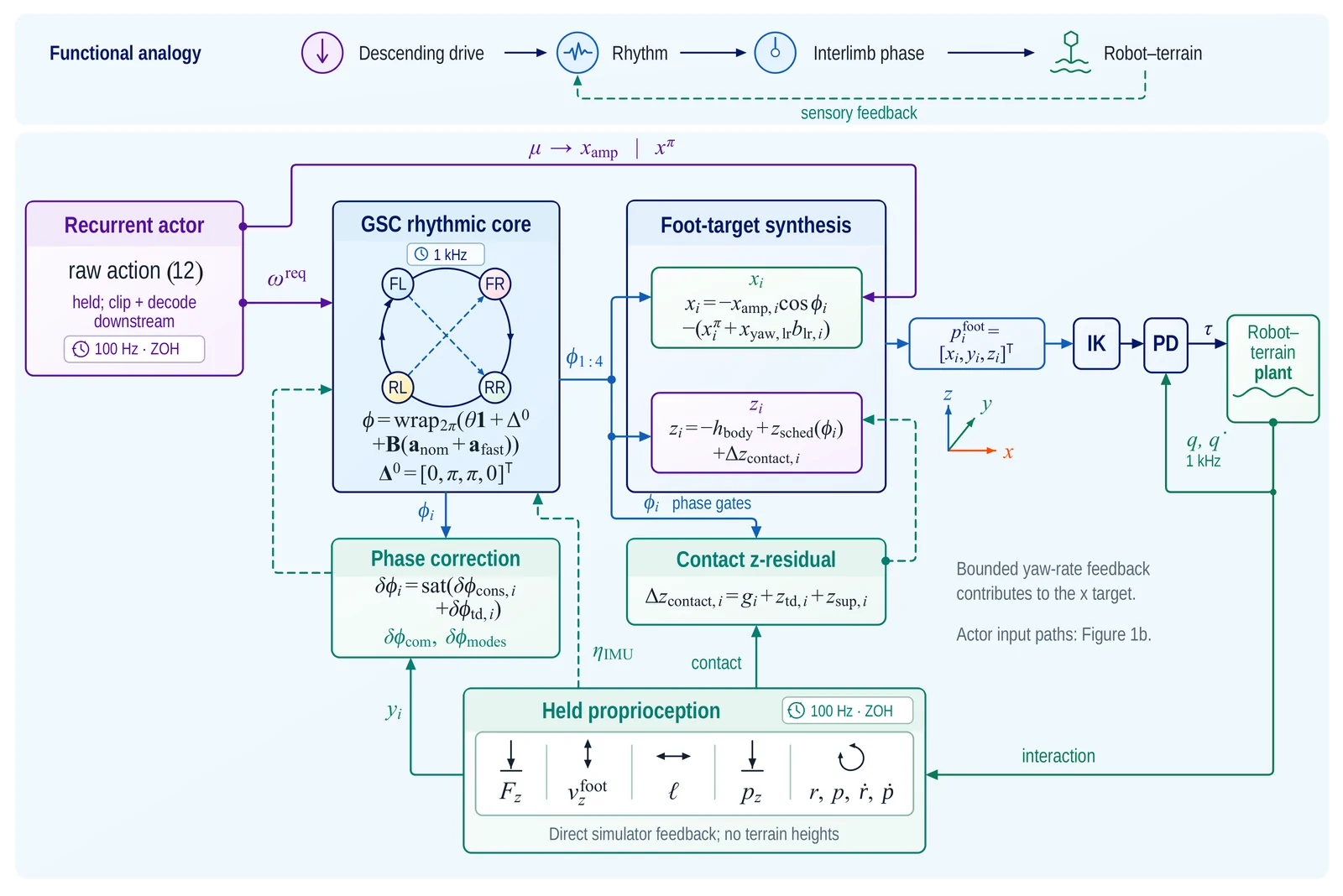
Biomimetic organization and signal flow of the GSC-CPG. The upper strip links descending drive, rhythm generation, interlimb coordination, robot–terrain interaction, and sensory feedback. Actor commands at 100 Hz use a zero-order hold (ZOH) across ten 1 ms controller updates. The rhythmic core generates coordinated leg phases; phase-conditioned proprioceptive innovation supplies bounded common/modal corrections, while contact residuals act on the vertical target. Analytic inverse kinematics (IK) and joint-space PD control close the loop. Terrain height is not a runtime input. *Leg labels:* FL, front left; FR, front right; RL, rear left; RR, rear right.

Figure 2 summarizes the biomimetic organization and implemented signal flow of the shared controller.

Leg order is [FL, FR, RL, RR] (front left, front right, rear left, and rear right, respectively). One common phase θ advances the cycle, while three modal coordinates describe front–hind, left–right, and diagonal timing. The four leg phases are

(2) $$\phi ={\mathrm{wrap}}_{2\pi }\;\left[\theta 1+\Delta^{0}+B\left(a_{\mathrm{nom}}+a_{\mathrm{fast}}\right)\right],\;\Delta^{0}={\left[0,\pi ,\pi ,0\right]}^{⊤}.$$

The modal basis is

(3) $$B=\frac{1}{2}\left[\begin{array}{ccc} 1 & 1 & 1\\ 1 & -1 & -1\\ -1 & 1 & -1\\ -1 & -1 & 1 \end{array}\right],\;a={\left[a_{fh},a_{lr},a_{d}\right]}^{⊤}.$$

The chart is centered on trot; its three modal axes identify the symmetric pace, bound, and pronk landmarks, while the implemented controller remains within the trot-centered bounds in Table 1.

Persistent and transient corrections occupy separate states. A slow nominal state acts only along the front–hind mode, while the fast state uses the remaining modal authority. Their combined excursion is clipped to the bounds in Table 1.

#### 2.2.2. Bounded Phase-Conditioned Sensory Correction

The controller uses phase to describe both cycle timing and the expected proprioceptive pattern of each leg. Sensory deviations from this pattern provide a bounded correction to the phase.

For leg *i*, a normalized proprioceptive vector is compared with a fixed front- or rear-leg harmonic template:

(4) $$\begin{array}{cc} y_{i} & ={\left[F_{z,i}/s_{F},\;v_{z,i}^{\mathrm{foot}}/s_{v},\;\left(\ell_{i}-\ell_{0}\right)/s_{\ell }\right]}^{⊤},\\ {\hat{y}}_{i} & =C_{g(i)}\psi \left(\phi_{i}\right),\;\psi \left(\phi \right)={\left[1,\sin\phi ,\cos\phi ,\sin2\phi ,\cos2\phi \right]}^{⊤}. \end{array}$$

The normalization scales are $s_{F}=120$ N, $s_{v}=1.5$ m s^−1^, $\ell_{0}=0.30$ m, and $s_{\ell }=0.10$ m. Front- and rear-leg templates are fitted once by ridge regression and fixed across all methods. Let $r_{i}=y_{i}-{\hat{y}}_{i}$, $h_{i}=C_{g(i)}\partial \psi \left(\phi_{i}\right)/\partial \phi_{i}$, and $W_{i}={\left(R_{g(i)}+\varepsilon I\right)}^{-1}$. The regularized local least-squares correction and the combined touchdown correction are

(5) $$\begin{array}{cc} \delta \phi_{\mathrm{cons},i} & ={\mathrm{sat}}_{{\bar{\delta }}_{b}}\;\left(\frac{h_{i}^{⊤}W_{i}r_{i}}{h_{i}^{⊤}W_{i}h_{i}+\gamma^{-1}}\right),\\ \delta \phi_{i} & ={\mathrm{sat}}_{\bar{\delta }}\left(\delta \phi_{\mathrm{cons},i}+\delta \phi_{\mathrm{td},i}\right), \end{array}$$

where ${\mathrm{sat}}_{b}\left(x\right)=\mathrm{clip}\left(x,-b,b\right)$. The first line estimates the bounded phase shift that best reconciles the measured signals; the second adds the independently bounded touchdown-timing correction. All correction authorities are shared across methods and listed in Table 1.

The same common/modal chart projects cadence and sensory correction before the phase update:

(6) $$\begin{array}{cccc} \delta \phi_{\mathrm{com}} & =\frac{1}{4}1^{⊤}\delta \phi ,\; & \delta \phi_{\mathrm{modes}} & =B^{⊤}\delta \phi ,\\ \dot{\theta } & =\omega_{\mathrm{com}}+k_{\mathrm{com}}\delta \phi_{\mathrm{com}},\; & {\dot{a}}_{\mathrm{fast}} & =-\Lambda a_{\mathrm{fast}}+\omega_{\mathrm{modes}}+K\delta \phi_{\mathrm{modes}}+\eta_{\mathrm{IMU}}, \end{array}$$

where $\omega_{\mathrm{com}}=\frac{1}{4}1^{⊤}\omega^{\mathrm{cmd}}$ and $\omega_{\mathrm{modes}}=B^{⊤}\omega^{\mathrm{cmd}}$. The bounded inertial measurement unit (IMU) term maps body tilt and angular rate into front–hind and left–right modes. Controller states and foot targets are integrated at 1 kHz from measurements held between 100 Hz policy updates. The orthogonal chart separates common timing from relative phase, and the decay term returns transient modes toward the nominal gait.

#### 2.2.3. Foot-Target Synthesis and Contact Support

The controller uses a fixed 50/50 phase-domain swing–stance partition. Measured contact latches a per-leg ground reference and updates a filtered force reference during stable stance. A bounded early-stance state converts a positive force deficit into a vertical support residual:

(7) $$z_{i}^{\sup}=-{\bar{z}}_{\sup}s_{i},\;0\leq s_{i}\leq 1.$$

Contact hysteresis, together with phase and vertical-velocity gates, prevents reference updates outside stable support.

Sagittal and vertical foot targets are generated through separate pathways:

(8) $$\begin{array}{cc} x & =-x_{\mathrm{amp}}⊙\cos\phi -\left(x^{\pi }+x_{\mathrm{yaw},\mathrm{lr}}b_{lr}\right),\\ z & =-h_{\mathrm{body}}1+g+z_{\mathrm{sched}}\left(\phi \right)+z_{\mathrm{td}}+z_{\sup}. \end{array}$$

Here, $x_{\mathrm{amp}}$ sets sagittal excursion, $z_{\mathrm{sched}}$ defines phase-scheduled lift, and $z_{\mathrm{td}}$ and $z_{\sup}$ provide bounded contact responses; $b_{lr}=\frac{1}{2}{\left[1,-1,1,-1\right]}^{⊤}$. Cadence and phase correction shape sagittal motion through ϕ, whereas contact pathways act only on the vertical target. Table 1 lists the shared authority limits.

### 2.3. Privileged Teacher–Student Learning

#### 2.3.1. Training–Deployment Information Boundary

The 68-D actor observation combines body motion and projected gravity (9), velocity command (3), joint-position error and velocity (24), preceding clipped action (12), binary contact (4), and GSC-CPG state (16). During training, the teacher and critic additionally receive a yaw-aligned 12×5 terrain-height grid:

(9) $$o_{t}^{\mathrm{actor}}=o_{t}^{68},\;o_{t}^{\mathrm{priv}}=\left[o_{t}^{68},h_{t}^{60}\right]\in R^{128}.$$

The terrain block is never exposed to the student actor and is absent at deployment.

#### 2.3.2. Teacher, Student, and Training Objective

The teacher actor and the privileged critic each use a feed-forward multilayer perceptron with a 128-D input and three hidden layers with exponential linear unit activations and 512, 256, and 128 units, respectively. The student encodes its 68-D observation into 128 features, processes them with a 128-unit GRU, and predicts the 12-D Gaussian action mean through 256- and 128-unit layers [35]. The recurrent state is reset at episode termination. The following stages describe teacher training with proximal policy optimization (PPO), behavior cloning, and online student optimization.

##### Teacher PPO Training

The terrain-aware teacher was trained with PPO from random initialization using seed 1, without resuming a checkpoint. Training comprised 2500 updates in 4096 parallel environments, with 24 policy steps per rollout, giving 245,760,000 environment transitions. Each update used five learning epochs and four minibatches. Adam used an initial learning rate of $10^{-3}$, $\left(\beta_{1},\;\beta_{2}\right)=\left(0.9,\;0.999\right)$, $\epsilon =10^{-8}$, and zero weight decay.

The PPO clipping parameter was 0.2, the discount factor was 0.99, and the generalized advantage estimation (GAE) parameter was 0.95. Training used a clipped value loss with coefficient 1.0, entropy coefficient 0.01, and gradient norm limit 1.0. Within an update, the learning rate was divided by 1.5 when the mean Kullback–Leibler (KL) divergence exceeded 0.02 and multiplied by 1.5 when the divergence was positive but below 0.005. The learning rate was bounded by $\left[10^{-5},\;10^{-2}\right]$. The Gaussian exploration policy used a fixed unit standard deviation in each action component.

Teacher training used the Full GSC-CPG, the five reward terms in Table 2, and clean 128-D observations. The terrain comprised a 4×4 grid of 20×20 m cells sharing one random roughness realization, with heights in [−0.06, 0.06] m and no terrain curriculum. The horizontal and vertical mesh scales were 0.10 and 0.01 m. Forward commands were sampled from 0.6–1.2 m s^−1^ every 5 s; lateral and yaw commands were zero. Friction coefficients were randomized over [0.2, 1.5] and added base mass over [−1, 6] kg. Observation noise, external pushes, proportional–derivative (PD) randomization, and actuator-delay randomization were disabled. Episodes ended after 20 s or earlier termination.

The final teacher checkpoint at update 2500 was used for student learning and remained frozen throughout BC and online student training. All student seeds therefore shared one teacher and one BC dataset. Teacher and student actors used the same structured action and Full GSC-CPG.

##### Behavior-Cloning Data and Sequence Training

The frozen teacher collected 512,000 observation–action pairs in 512 environments over 1000 policy steps, using collection seed 7001. Its deterministic actions drove all rollouts. The dataset used independently generated rough-terrain cells with heights between −0.06 and 0.06 m. Forward commands were resampled every 5 s from 0.6–1.2 m s^−1^, with zero lateral and yaw commands. Collection started from an environment reset and continued through automatic resets after termination.

Observations, deterministic teacher action means, and termination masks were stored in time order. The student received only the 68-D terrain-height-free observation prefix. We assigned 410 complete environment sequences to training and 102 to validation, keeping every sequence within one set. The dataset contained teacher-driven rollouts only.

BC used batches of 64 environment sequences, processed in consecutive 128-step chunks. The loss was the mean squared error (MSE) across the 12 action components between the student mean and teacher means clipped to [−4, 4]. The preceding-action observation field was also clipped to this range during BC preprocessing.

The recurrent state was initialized at the start of each sequence batch, carried between consecutive chunks, and detached from the computation graph after each optimization step. After a terminal prediction, the termination mask reset the state of the affected environment. This retained temporal order within an episode while separating successive episodes.

Each BC seed started from a randomly initialized student model. Only the actor encoder, GRU, and action head were optimized; the privileged critic retained its random initialization. AdamW used a learning rate of $10^{-3}$, weight decay $10^{-5}$, and gradient norm limit 1.0. Training used a 200-epoch limit and patience of 25 epochs. Validation MSE had to decrease by more than $10^{-5}$ to update the retained model. All three runs completed 200 epochs. The selected epochs were 190, 190, and 200 for seeds 1–3, with validation MSEs of 0.013727, 0.013267, and 0.013152. The three runs used the same dataset and validation split.

##### Online Student Objective

After BC, each recurrent student is optimized with PPO [36] and scheduled teacher supervision. The actor objective is

(10) $$\begin{array}{c} L_{\mathrm{actor}}\left(k\right)=c_{\mathrm{PPO}}\left(k\right)L_{\mathrm{PPO},\mathrm{actor}}+\lambda_{T}\left(k\right)\;E_{t}\;\left[\frac{{∥\mu_{\theta }\left(o_{0:t}^{68}\right)-a_{t}^{\mathrm{teach}}∥}_{2}^{2}}{n_{a}}\right],\\ \;a_{t}^{\mathrm{teach}}=C_{4}\left[\pi_{T}\left(o_{t}^{128}\right)\right]. \end{array}$$

Here, $n_{a}=12$, and $\pi_{T}$ denotes the deterministic raw teacher action mean. The teacher target is clipped by $C_{4}$, whereas the student mean $\mu_{\theta }$ remains unclipped. The second term is therefore an MSE supervision loss averaged over the action components.

Online training began with 50 critic warm-up updates while the actor was fixed, followed by 250 joint updates. At joint update k=0,…,249, $\lambda_{T}\left(k\right)=0.5-0.45k/1000$ and $c_{\mathrm{PPO}}\left(k\right)=0.1k/1000$. The teacher-loss coefficient thus ranged from 0.5 to 0.38795, and the PPO coefficient ranged from zero to 0.0249. These runs used the first 250 updates of the schedules’ 1000-update spans.

#### 2.3.3. Compared Training Methods and Runtime Information Boundary

The teacher and online student PPO procedures use the same five-term reward and a critic with clean 128-D privileged observations. The student methods differ in actor architecture, teacher supervision, and actor observations (Table 3). Their common reward is $r_{t}=\Delta t\sum_{m}w_{m}\rho_{m,t}$.

The reward is applied once per 0.01 s control interval, and the action-rate term uses $a_{t}^{100}$. No explicit phase-tracking reward is used; phase adaptation remains in the fixed sensory and contact pathways.

Unless otherwise stated, the proposed student denotes the teacher-guided GRU trained with observation corruption.

All online student runs used Adam and 512 environments, with a rollout horizon of 24 steps, PPO clipping 0.2, γ=0.99, GAE λ=0.95, and a clipped value loss with coefficient 1.0.

The feed-forward (FF) and GRU baselines trained from random initialization for 1500 updates, equivalent to 18,432,000 environment transitions. Both used adaptive KL scheduling with target 0.01, entropy coefficient 0.01, and initial action standard deviation 1.0. The FF baseline used learning rate $10^{-3}$, five optimization epochs, four minibatches, and gradient norm limit 1.0. The GRU baseline used learning rate $3\times 10^{-4}$, one epoch, one minibatch, and gradient norm limit 0.5.

Students initialized by BC trained online for 300 updates, including warm-up, giving 3,686,400 environment transitions. Each run loaded the complete BC model, including the untrained critic, and started with fresh optimizer and PPO state. Actor and critic learning rates were fixed at $10^{-6}$ and $10^{-4}$, respectively. Training used zero entropy coefficient, initial action standard deviation 0.2, one optimization epoch, one minibatch, and gradient norm limit 0.5. All online comparisons used the final checkpoint at the stated budget, without selection on test performance. The five-method comparisontherefore evaluates complete training procedures, including these differences in initialization and optimization.

The main experiment used three matched pairs of recurrent student policies. Each pair started from the same BC checkpoint and used the same actor and critic architectures, environment, independent and identically distributed (IID) noise profile, optimizer settings, 300-update budget, and PPO coefficient schedule. The guidance-off condition set the teacher-loss coefficients to zero. This design evaluates the additional contribution of continued teacher guidance after BC initialization.

**Figure 3:**
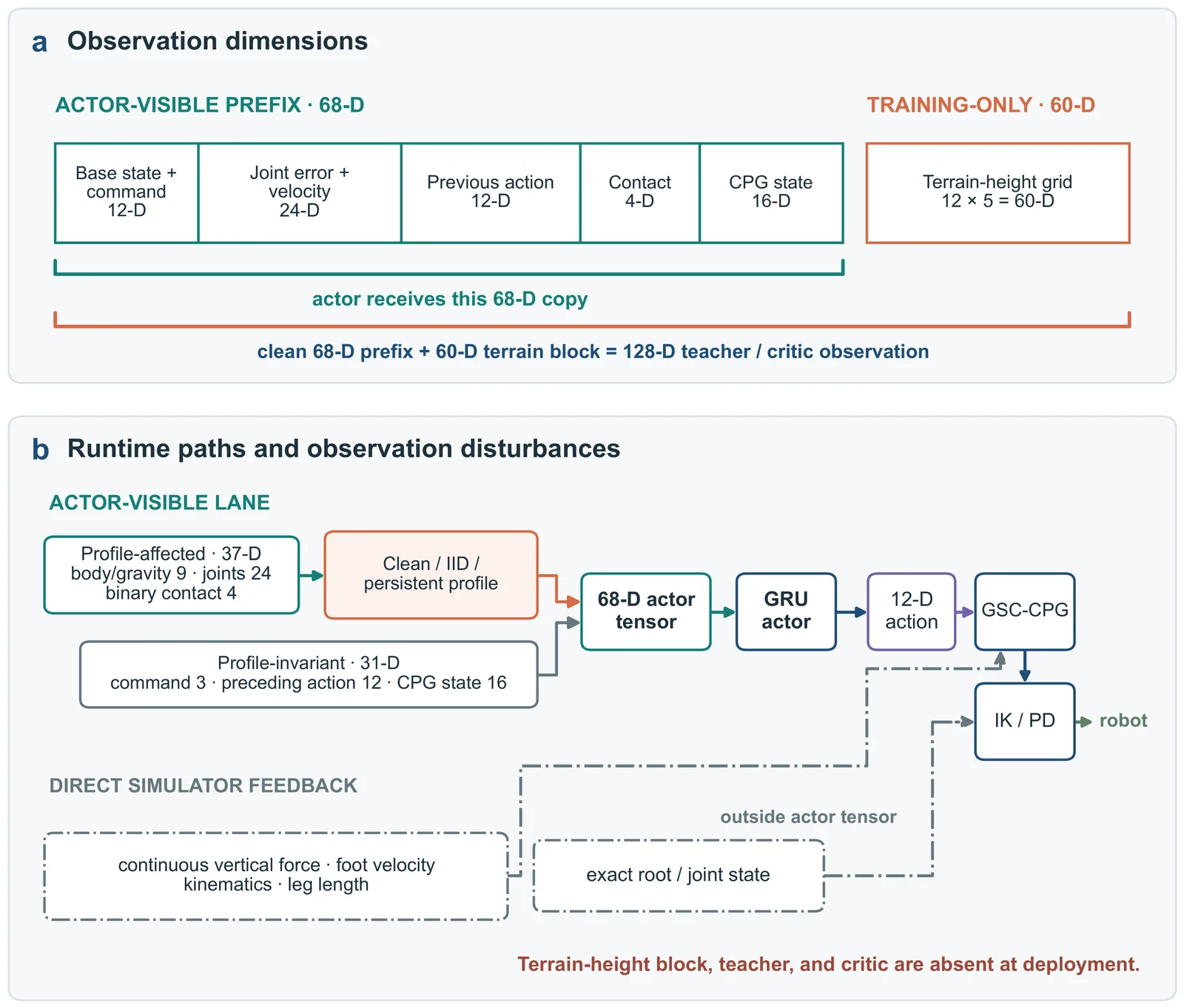
Observation composition and training–deployment paths. (**a**) The deployed actor receives a 68-D proprioceptive and rhythmic state; the 60-D terrain grid augments only the privileged training tensor. (**b**) Actor-visible fields and downstream feedback are separate paths. The observation profile affects 37 actor-visible state and contact fields; commands, action history, and CPG state account for 31 unaffected fields. Deployment retains the actor and fixed controller with direct simulator feedback, but no terrain heights, teacher, or critic.

Observation disturbances are applied to the actor input. The fixed downstream controller receives direct simulator contact, kinematic, root, and joint feedback through separate paths (Figure 3).

### 2.4. Simulation Experiments

Experiments use the Unitree Go2 model in Isaac Gym [37]. Physics and GSC-CPG run at 1 kHz, and the actor updates at 100 Hz. Each actor output is held across ten 1 ms GSC-CPG, inverse-kinematics, and joint-PD updates. Joint feedback refreshes each physics step; root, rigid-body, and contact measurements refresh at the actor boundary and are held between refreshes. Each 20 s trial commands straight-line motion at 0.6–1.2 m s^−1^. Policies are trained on randomized ±6 cm rough terrain; evaluation additionally covers held-out ±8 cm roughness and an unseen 5° uphill slope.

Terrain heights are converted to a fixed triangle mesh and contacted by the rigid-body PhysX model; the terrain does not deform or move. The simulation uses one substep per 1 ms step, the temporal Gauss–Seidel solver, four position iterations, zero velocity iterations, contact offset 0.01 m, and rest offset zero. These settings define the numerical rigid-contact model used in all reported simulations.

Five student methods are evaluated under clean actor observations, IID measurement noise, and persistent disturbances. The matched guidance comparison uses the paired initializations and training settings described above. The 5° slope evaluation uses three trained policies per method, three evaluation seeds, and 128 parallel environments per cell. This gives 1152 launched environment trajectories and a total exposure of 384 robot-minutes per method.

Controller tests compare Backbone, Support, and Full configurations. Backbone contains phase innovation, touchdown timing, and ground-reference latching; Support adds bounded vertical contact responses; Full additionally updates the force reference during stable stance.

#### 2.4.1. Actor-Observation Disturbances

The clean profile leaves simulator observations unchanged. Both disturbance profiles affect body velocity, projected gravity, joint state, and binary contact, comprising 37 actor fields. Commands, preceding action, and CPG state comprise the remaining 31 fields and are unchanged. Table 4 gives the per-component analog disturbances in physical units. Linear velocity, angular velocity, joint position, and joint velocity are scaled by 2, 0.25, 1, and 0.05, respectively, when forming the actor observation. Projected gravity is renormalized to unit length after perturbation.

IID noise is sampled independently at each actor step, without bias, drift, delay, or dropout. Its contact false-positive and false-negative probabilities are 0.005 and 0.01 per foot per step. Persistent noise follows a first-order autoregression with coefficient 0.8 and innovation standard deviation (SD) $\sigma \sqrt{1-0.8^{2}}$. Bias is drawn independently for each environment and held for the episode; noise and drift states start at zero after reset.

For persistent disturbances, a common sensor delay of 1–3 actor steps is drawn uniformly per environment at reset. Independent stepwise jitter of −1, 0, or 1 step gives an effective delay of 0–40 ms. The delayed contact signals additionally receive a uniform 0–20 ms touchdown delay and false-positive/negative probabilities of 0.01/0.03. Velocity, IMU, and contact dropouts start with probabilities 0.002, 0.001, and 0.002 per eligible environment step, respectively. Each holds the last output for a uniformly drawn 1–5 steps (10–50 ms). IMU dropout jointly holds angular velocity and gravity; joint signals do not undergo dropout. Delay precedes analog perturbation, contact errors, and dropout. Histories are initialized from the reset observation, and no dropout begins on that step. The profiles are synthetic stress tests rather than calibrated hardware sensor models. The privileged tensor and downstream controller feedback remain unperturbed.

#### 2.4.2. Outcome Definitions

A failure is a physical fall or terrain-boundary exit before the 20 s horizon; timeout is not a failure. Failure rate is the event count divided by accumulated robot exposure, expressed per robot-minute. Environments reset after termination and continue contributing to the fixed-duration evaluation. “Fall” refers only to the physical-fall category. Tracking root-mean-square error (RMSE) is $\sqrt{\langle ∥c_{xy}-v_{xy}{∥}_{2}^{2}\rangle }$, and tilt-proxy root mean square (RMS) is $\sqrt{\langle ∥g_{xy}^{\mathrm{proj}}{∥}_{2}^{2}\rangle }$. The tilt proxy is dimensionless. Both metrics use unperturbed simulator state; angle brackets denote averaging over environment-time samples. Action saturation is the fraction of raw actor components exceeding magnitude 4.

Raw action-change RMS is the root mean square of the difference between consecutive raw actions, averaged across the 12 action components and all environment-time samples. Execution-equivalent action-change RMS applies $C_{4}$ to both actions before differencing, without division by 4. Both describe variation in action units per 10 ms update, rather than a time derivative. The previous action is set to zero at initialization and episode reset, so episode-start changes are included. These diagnostics characterize policy-output variation; they are not measurements of motor effort or wear.

#### 2.4.3. Replication and Statistical Analysis

The replication unit is the separately trained student policy. The main comparison crosses three paired student training seeds with evaluation seeds 4011–4013 and four fixed conditions: two roughness levels and two disturbance profiles. Each of the 36 cells per method contains 128 environments evaluated for 2000 actor steps. Each pair shares its BC initialization and is evaluated with matching evaluation seeds, terrain, and disturbance conditions. Parallel environments, time steps, and reset episodes contribute exposure and metric samples, not additional training replicates.

**Figure 4:**
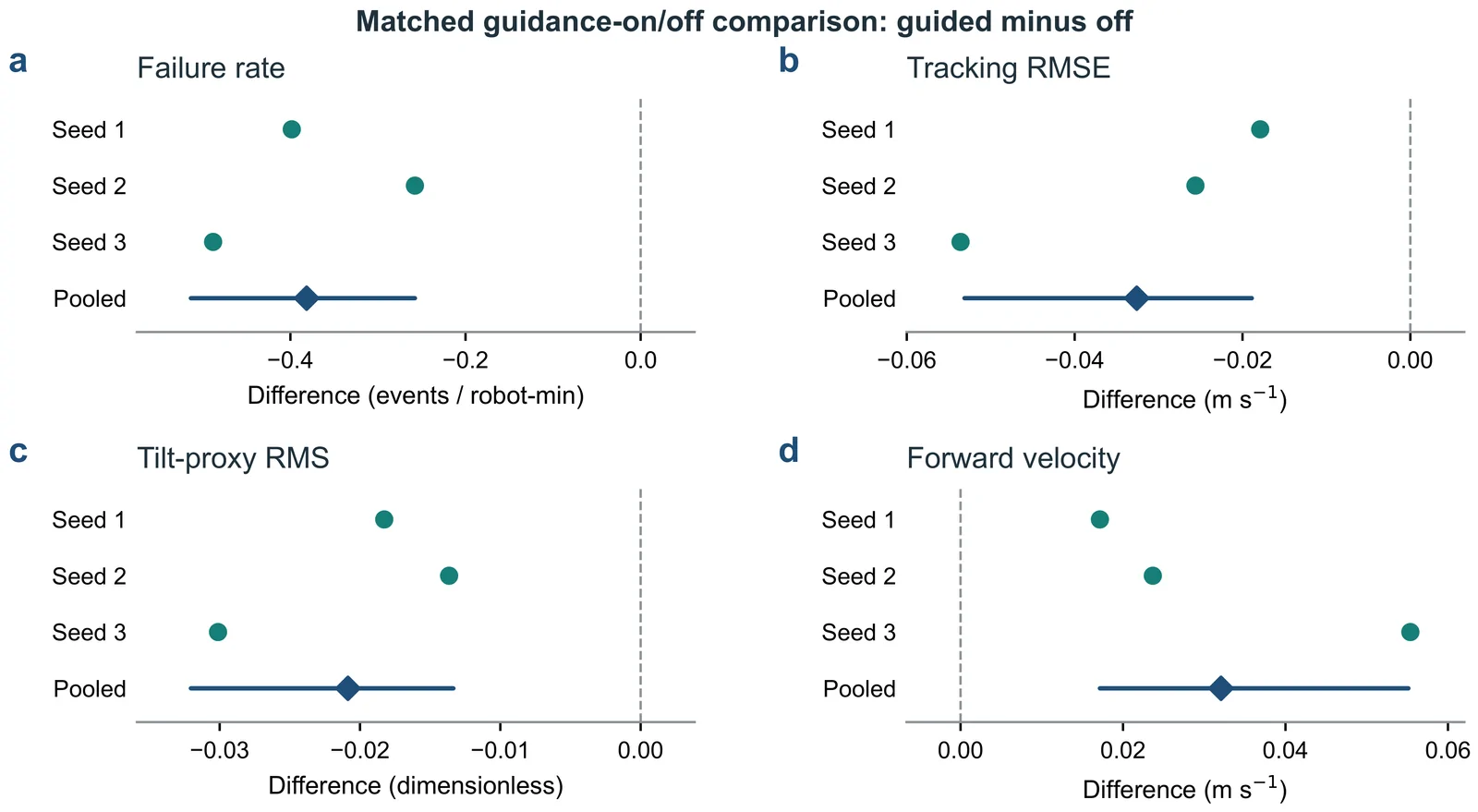
Matched guidance-on/off contrasts after BC initialization. (**a**) Failure rate; (**b**) tracking RMSE; (**c**) tilt-proxy RMS; (**d**) mean forward velocity. Each circle is the guided-minus-off contrast for one paired student training seed, pooled over three evaluation seeds and the four fixed terrain–disturbance conditions. Diamonds and horizontal bars show the pooled contrast and its endpoint-wise 95% crossed-bootstrap interval. The dashed line denotes no difference. Negative values favor guidance for failure rate, tracking RMSE, and tilt-proxy RMS; positive values favor guidance for forward speed. The three training seeds share a frozen teacher and BC dataset.

Two RMS aggregation conventions are used. If cell *c* contains $n_{c}$ samples and has RMS $R_{c}$, the matched analysis uses $R_{\mathrm{pooled}}=\sqrt{\sum_{c}n_{c}R_{c}^{2}/\sum_{c}n_{c}}$. This reconstructs the RMS across pooled samples and is used in Table 5 and Figure 4. The five-method comparison instead averages cell RMS values, ${\bar{R}}_{\mathrm{cell}}=\sum_{c}n_{c}R_{c}/\sum_{c}n_{c}$. Table 6 reports the mean and sample SD of three training-seed summaries using this convention. Its evaluation seeds are 110–112, with the same four terrain–disturbance conditions. The two RMS summaries are not interchangeable. Forward speed and saturation use sample-weighted means; failure rates pool event counts and exposure.

Matched effects are guided-minus-guidance-off differences. Their 95% intervals use 20,000 crossed paired bootstrap resamples. Three training-seed labels and three evaluation-seed labels are sampled independently with replacement and crossed. Identical multiplicities are applied to both methods, while all four conditions remain fixed. The 2.5th and 97.5th percentiles define endpoint-wise, non-simultaneous intervals (bootstrap seed 20260814; Python 3.8.20, NumPy 1.24.3). The intervals describe variation within the sampled student and evaluation seeds, conditional on the shared teacher and BC dataset. We report the individual training-seed contrasts alongside them and interpret the results as effect estimates for this design, rather than population-level significance claims. In the five-method comparison, identical seed labels do not imply shared initialization between from-scratch and BC-initialized methods. The controller comparison uses one separately trained policy per configuration and three shared evaluation seeds. The sensory-response test varies prescribed inputs and initial phases, independently of a closed-loop robot simulation.

## 3. Results

### 3.1. Contribution of Scheduled Teacher Guidance

Continued teacher guidance reduced the pooled failure rate from 1.479 to 1.097 events per robot-minute in the matched student comparison (Table 5). Tracking error, body tilt, and action saturation decreased, while forward speed increased. All three paired training runs showed the same direction of change for these outcomes (Figure 4). Both raw and execution-equivalent action-change RMS increased, indicating that the locomotion improvements were accompanied by greater temporal variation in the policy outputs.

Supplementary Video S4 shows the matched students on the same ±6 cm rough-terrain realization under clean actor observations and a $0.8\;m\;s^{-1}$ command. Both run without the teacher or terrain-height input at deployment.

### 3.2. Transfer to an Unseen Uphill Slope

Without retraining or terrain-height input, the three guided policies incurred no physical falls during the 5° slope evaluation. The guidance-off policies incurred nine physical falls under the same 384 robot-minute exposure. Neither method incurred a boundary exit.

**Figure 5:**
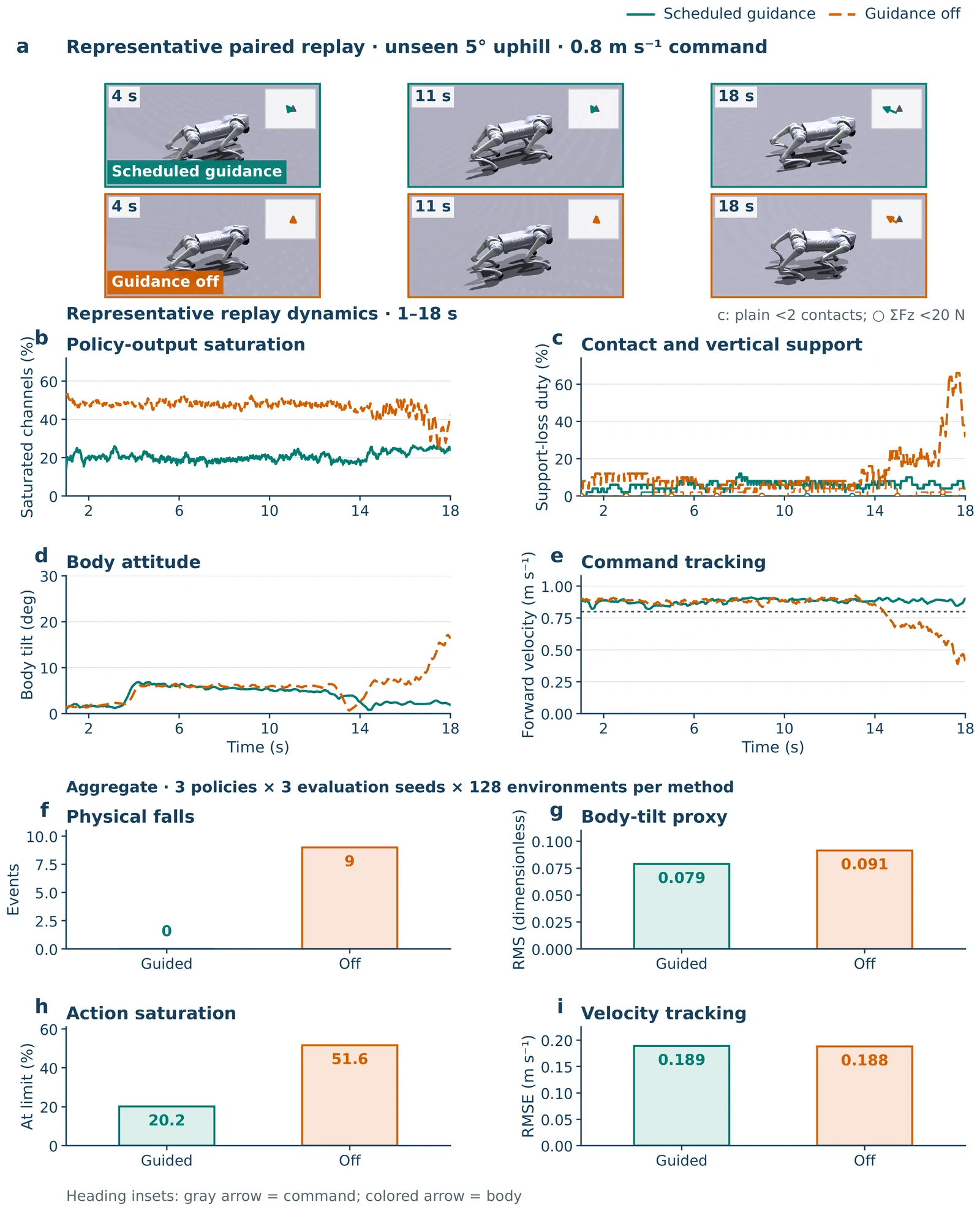
Matched replay and aggregate validation on an unseen 5° uphill slope. (**a**) Synchronized frames at 4, 11, and 18 s under a fixed 0.8 m s^−1^ command. The camera follows base translation and yaw while remaining world-up; each inset compares body and commanded uphill headings. (**b**) Policy-output saturation. (**c**) Support loss. (**d**) Body tilt. (**e**) Velocity tracking. Panels (**b**–**e**) span 1–18 s. Both replays reached 20 s and are shown in Supplementary Video S5. (**f**) Physical falls. (**g**) Tilt-proxy RMS. (**h**) Action saturation. (**i**) Velocity-tracking RMSE. Panels (**f**–**i**) summarize aggregate evaluation with three trained policies, three evaluation seeds and 128 parallel environments per cell. Both methods share the GSC-CPG and use paired BC initializations; training details are given in Section 2.3.

Both policies reached the 20 s horizon in the representative paired replay. The guided policy maintained more continuous contact support, lower body tilt, and closer command tracking; the guidance-off policy developed greater support loss, tilt, and velocity error after approximately 14 s. In the separate aggregate evaluation, guidance reduced body tilt by 13.8% and action saturation by 60.9%, with similar velocity tracking and forward speed (Figure 5).

### 3.3. Comparison of Complete Training Procedures

The guided recurrent controllers had lower pooled failure rates and errors than the evaluated BC and from-scratch baselines under actor-observation disturbances (Table 6). The table compares complete training procedures with different initialization, budgets, and optimizer settings. Among the guided students, clean-observation training produced slightly better pooled outcomes than corruption training; these results did not demonstrate an additional benefit from corruption training under the evaluated settings.

The from-scratch no-teacher GRU saturated all evaluated action components and moved backward on average, indicating unsuccessful optimization under that training configuration. Because the initialization and optimization settings differ across these procedures, the matched comparison in Section 3.1 provides the more direct assessment of continued teacher guidance.

Under clean observations, the proposed student remained close to the terrain-aware teacher. At ±6 cm, their respective failure rates were 0.096 and 0.086 events per robot-minute and their forward speeds were 0.715 and 0.732 m s^−1^. At the held-out ±8 cm roughness, the corresponding values were 1.151 and 0.992 events per robot-minute and 0.505 and 0.523 m s^−1^.

### 3.4. Generalization to Held-Out Roughness

**Figure 6:**
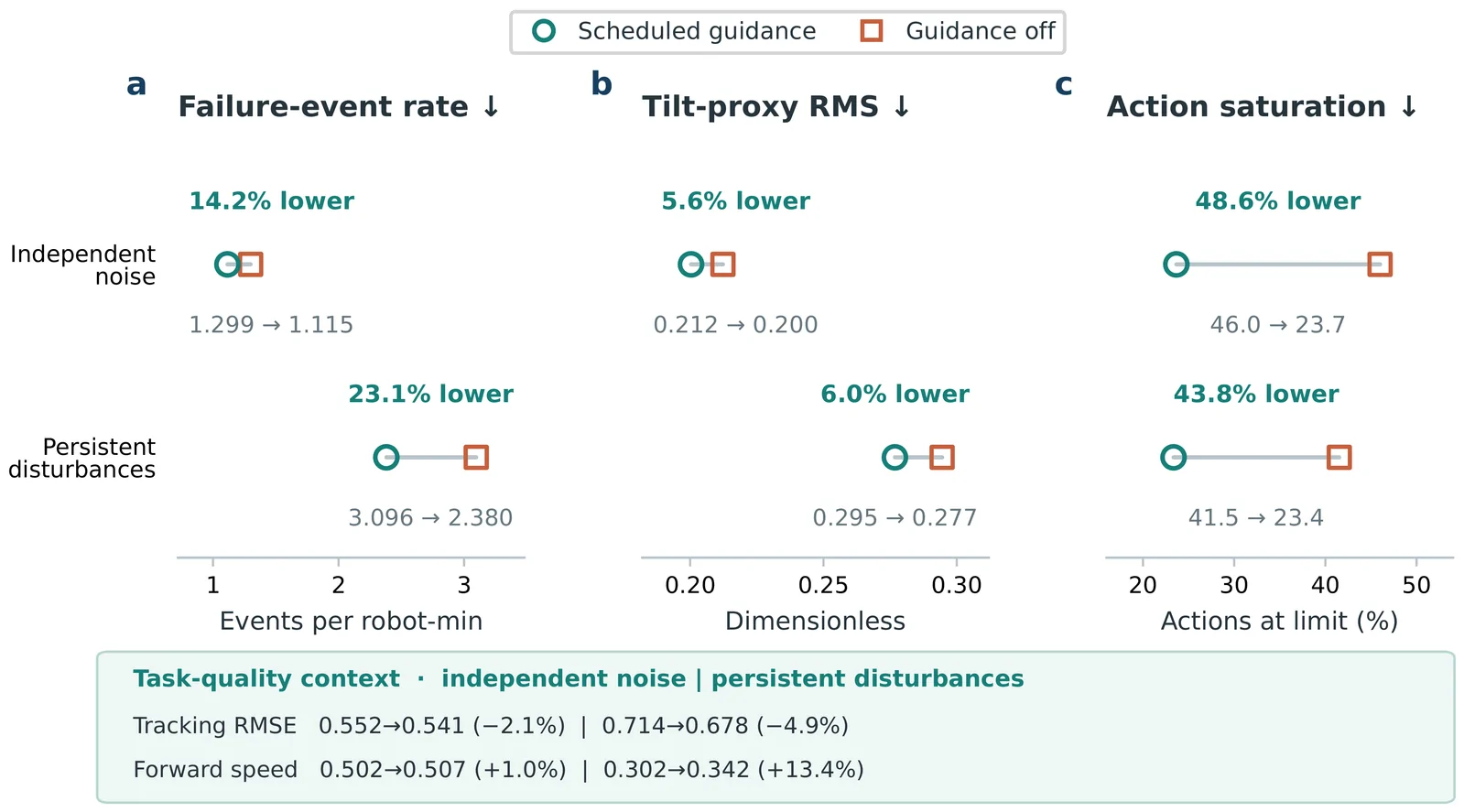
Matched evaluation on the held-out ±8 cm random rough terrain, beyond the ±6 cm training roughness. Panels compare scheduled-guidance and guidance-off training under independent noise and persistent disturbances: (**a**) failure-event rate, (**b**) body-tilt RMS, and (**c**) action saturation. Downward arrows in panel headings indicate that lower values are better. Arrows point from the guidance-off value to the scheduled-guidance value; the lower strip summarizes velocity tracking and forward speed. Values pool three BC-paired training seeds and three evaluation seeds within each condition; the panels report descriptive condition-specific estimates. Both recurrent policies are terrain-height-blind and teacher-free at deployment.

At the held-out ±8 cm roughness, scheduled-guidance training retained its advantage over the matched guidance-off policy under both independent noise and persistent disturbances (Figure 6). Failure rate decreased by 14.2% and 23.1%, body-tilt RMS by 5.6% and 6.0%, and action saturation by 48.6% and 43.8%, respectively. Tracking error was also lower while forward speed was preserved or increased.

### 3.5. Matched Dynamic Response on Rough Terrain

**Figure 7:**
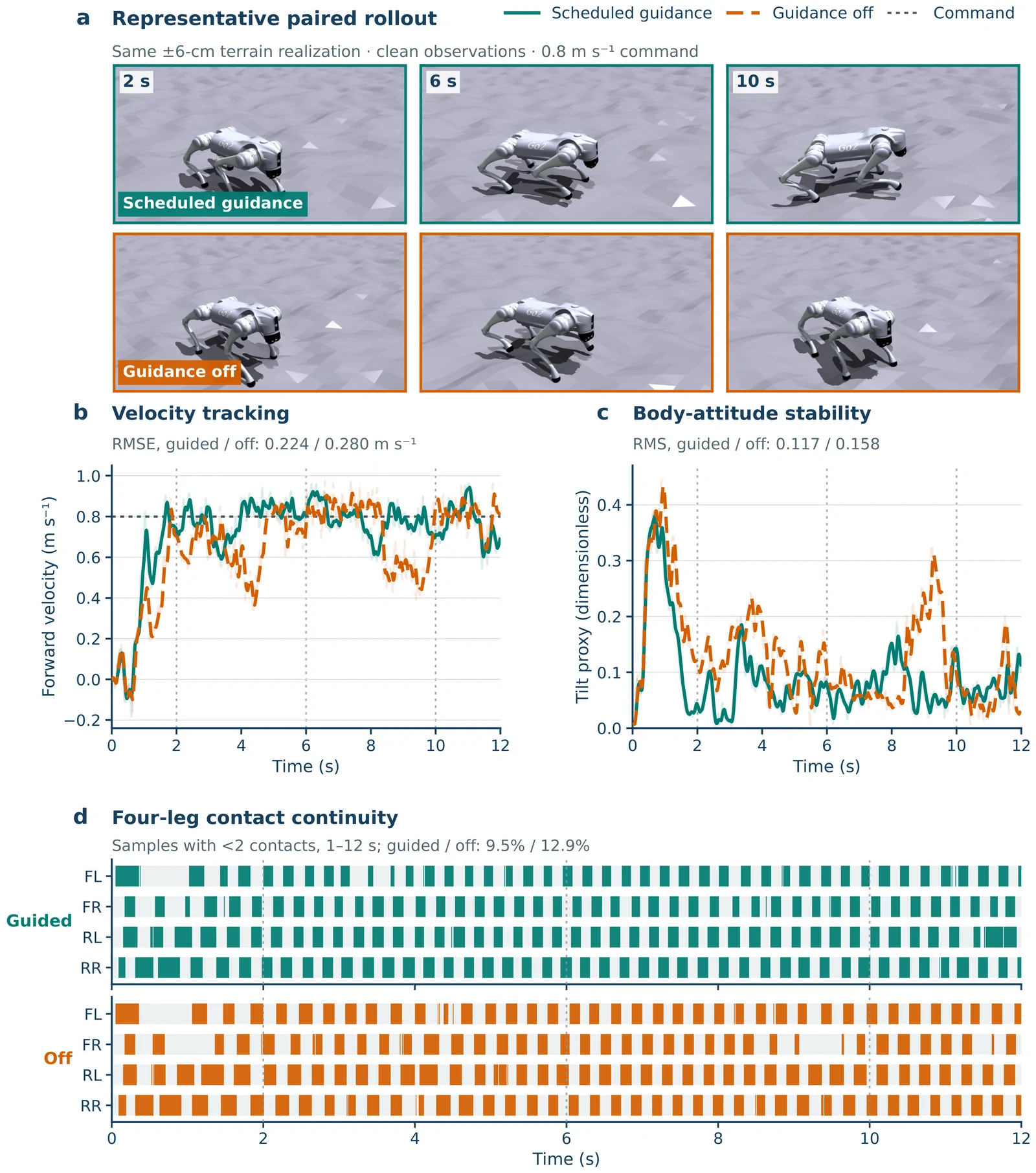
Matched rough-terrain response of the recurrent students. (**a**) Synchronized frames of the students trained with scheduled guidance and with guidance disabled on the same ±6 cm terrain realization. (**b**) Forward velocity relative to the 0.8 m s^−1^ command. (**c**) Body-tilt proxy derived from projected gravity. (**d**) Measured four-leg contact sequences. Both actors receive clean proprioceptive observations and operate without the teacher or terrain-height input at deployment. Supplementary Video S4 provides the complete comparison.

Figure 7 and Supplementary Video S4 illustrate the time-resolved responses in a representative matched rollout on the ±6 cm terrain under clean actor observations. Across this 12 s replay, scheduled guidance increased mean forward speed from 0.646 to 0.719 m s^−1^, reduced velocity-tracking RMSE from 0.280 to 0.224 m s^−1^, and reduced body-tilt RMS from 0.158 to 0.117. Between 1 and 12 s, the fraction of samples with fewer than two measured contacts decreased from 12.9% to 9.5%.

**Figure 8:**
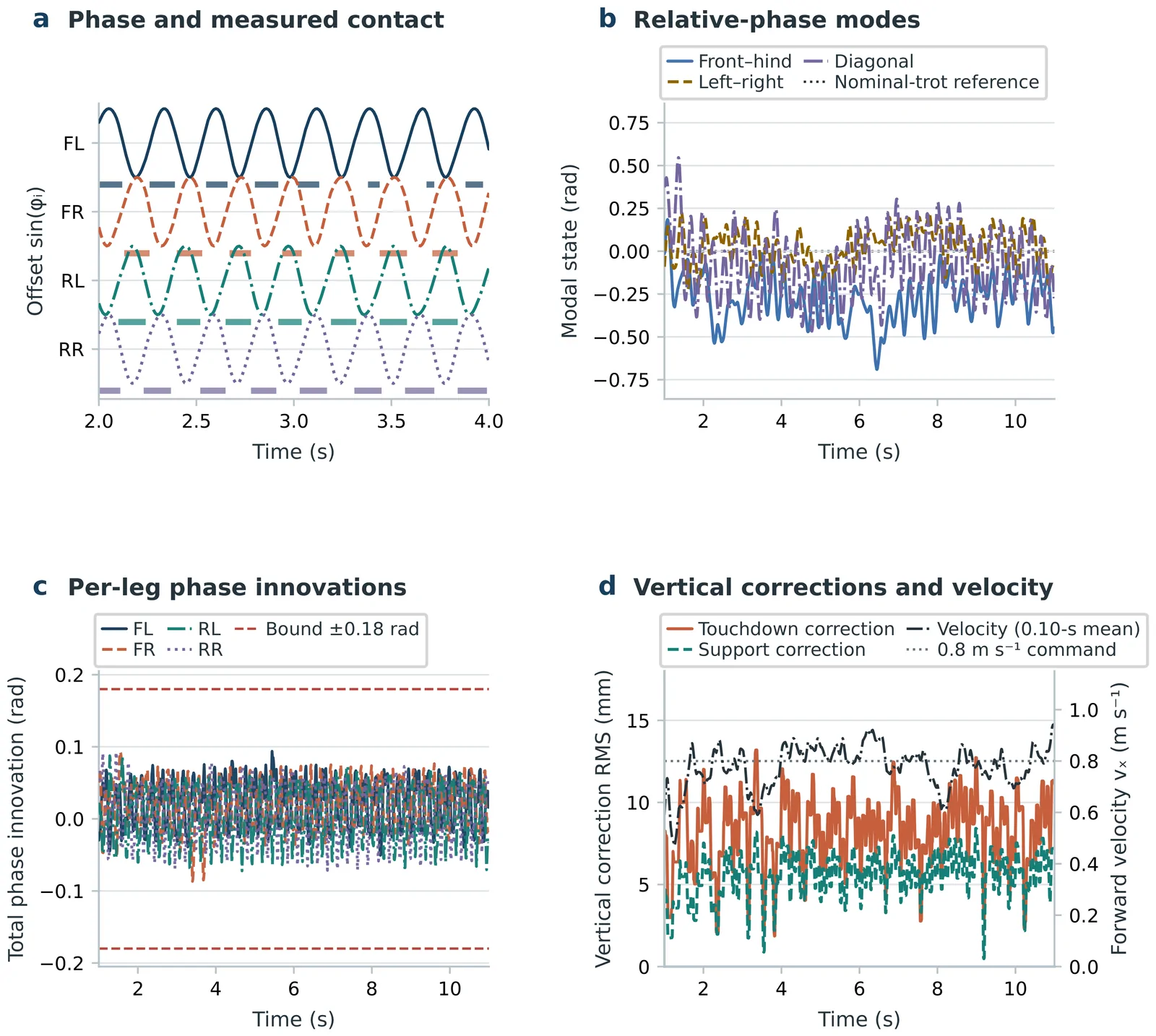
Controller activity in a representative guided-controller rollout under the ±6 cm clean-observation condition. (**a**) Phase-cycle signal and measured contact. (**b**) Relative-phase modal states. (**c**) Bounded per-leg phase innovations; their unsmoothed mean and maximum magnitudes were 0.097 and 0.178 rad. (**d**) Vertical touchdown/support correction and forward velocity. Panels show causal moving means; leg styles are shared between (**a**,**c**).

A separate guided-controller rollout under the same roughness, observation, and command condition exposes the internal GSC-CPG response in Figure 8.

The guided-controller trace sustained 0.722 m s^−1^ forward motion while phase and vertical corrections remained active and bounded.

### 3.6. GSC-CPG Phase Reconfiguration

**Figure 9:**
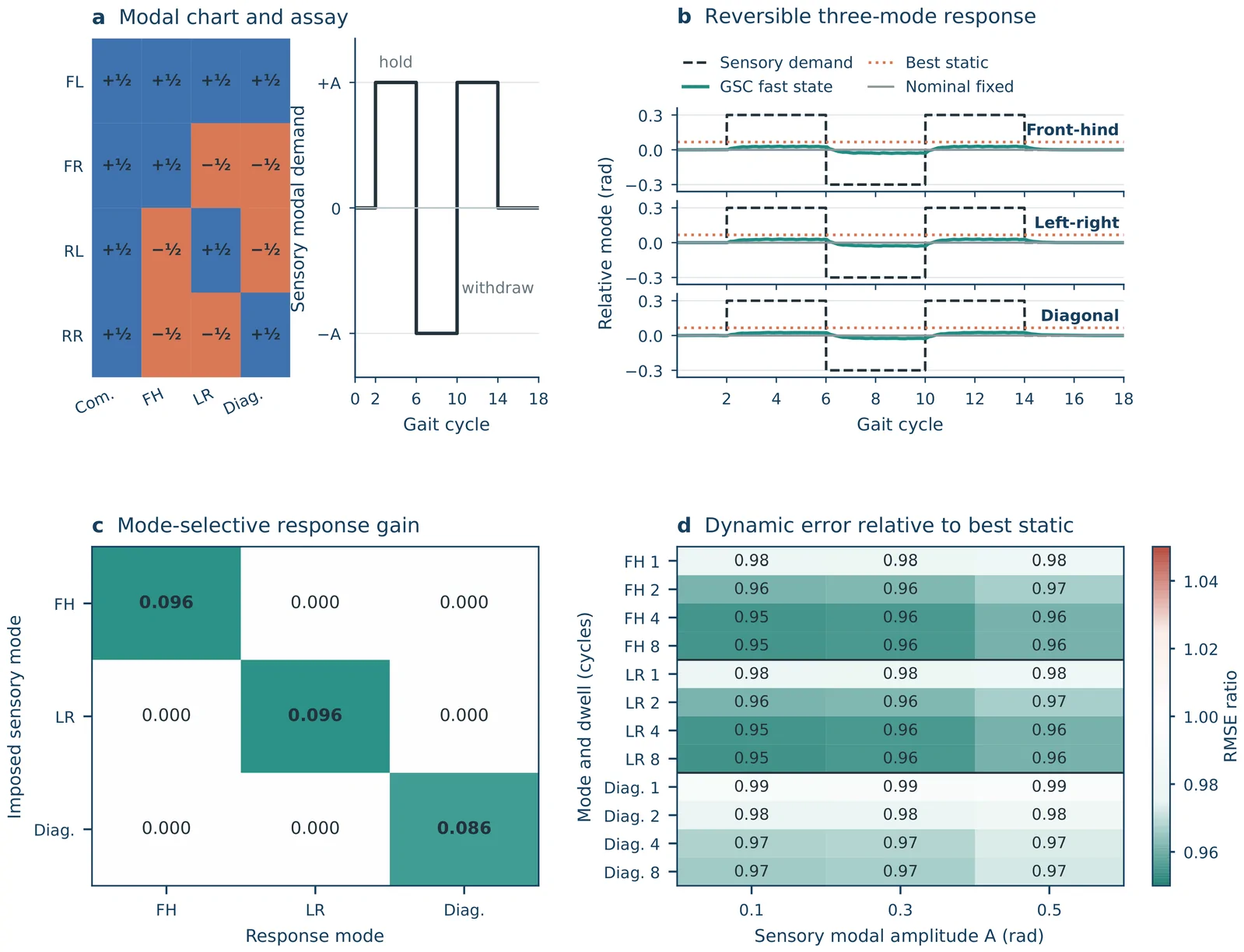
Numerical phase-response test of the GSC-CPG. (**a**) Common (Com.) and modal coordinates and prescribed 0→+A→−A→+A→0 timing sequence. (**b**) Front–hind (FH), left–right (LR), and diagonal (Diag.) responses (A=0.30 rad, four-cycle dwell, zero initial phase). Nominal fixed trot stays at zero; the best-static setting, selected for the complete input sequence, stays constant. (**c**) Signed modal gains, summarized over 12 initial phases and both input signs at A=0.30 rad and four-cycle dwell. (**d**) GSC/best-static phase-shape RMSE ratios across three amplitudes and four dwell times per mode. Ratios below one favor online reconfiguration.

The prescribed-input component test examines how the GSC-CPG responds when the sensory timing demand changes. Under constant demand, its median phase-shape RMSE was close to the best-static setting (0.069 versus 0.065 rad). Across the changing-demand sequences, the GSC-CPG had modestly lower error, with dynamic-error ratios of 0.955–0.994 across the tested modes, amplitudes, and dwell times. The ratio was 0.956 with correlated template noise (Figure 9). The static comparator was selected with knowledge of the complete prescribed sequence.

The phase response followed the imposed mode and returned toward the nominal gait after the input was withdrawn. The largest off-axis gain remained below $2\times 10^{-5}$, and 1.25% of raw innovations reached the base clip, showing mode-selective, reversible responses within the tested input range.

### 3.7. Contact and Support Regulation

**Figure 10:**
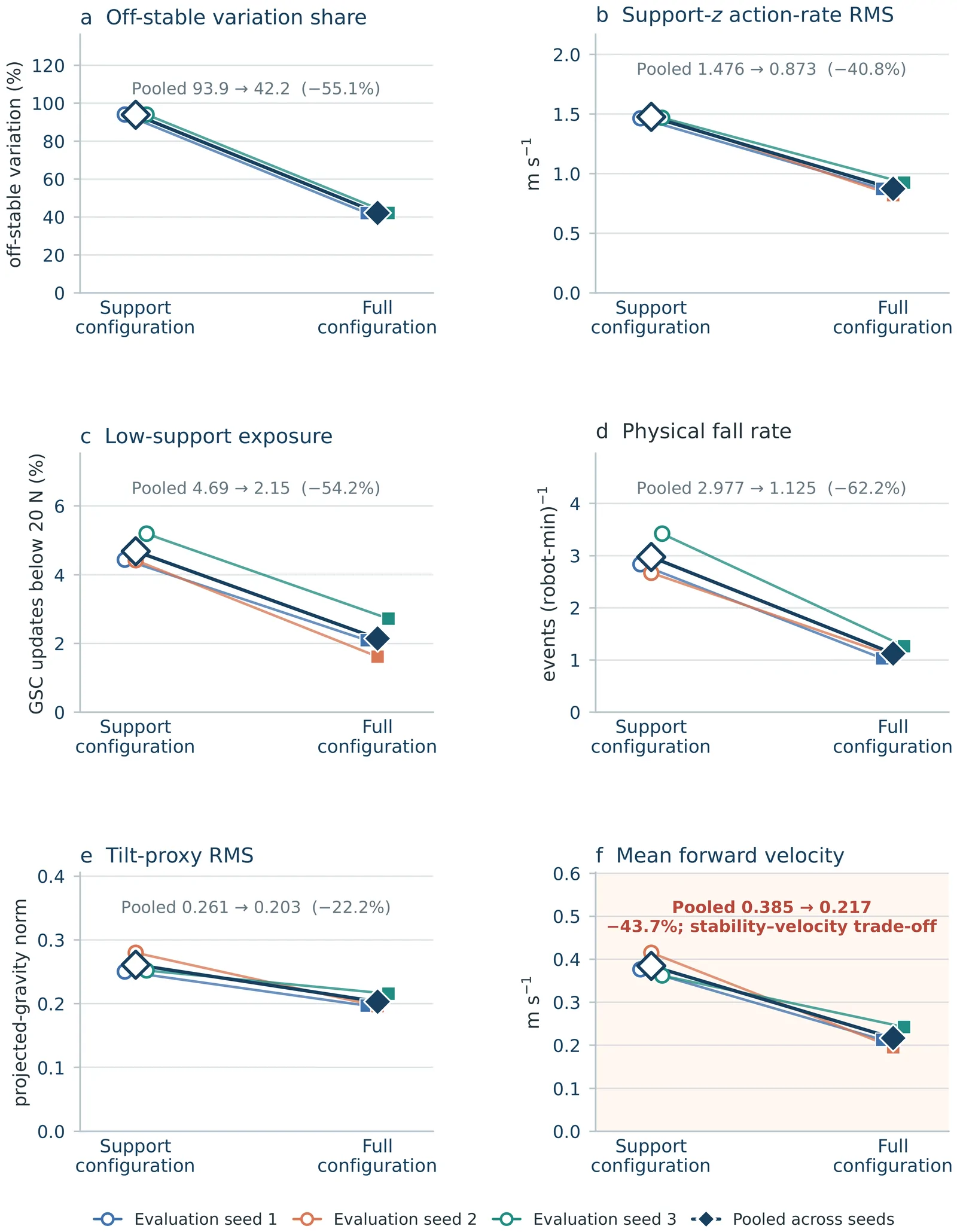
Support–Full comparison within the GSC-CPG component evaluation. One separately trained policy is used per configuration. Lines connect shared evaluation seeds 2101–2103, with 128 environments and 2000 actor steps per cell; diamonds show pooled values. (**a**) Off-stable share of reference variation. (**b**) Support-*z* action-rate RMS. (**c**) Low-support exposure below 20 N. (**d**) Physical-fall rate; no boundary exits occurred in these evaluations. (**e**) Tilt-proxy RMS. (**f**) Mean forward velocity.

The Full configuration had lower support variation, body tilt (0.203 versus 0.261), and physical-fall rate (1.13 versus 2.98 events per robot-minute) than Support. Mean forward speed decreased from 0.385 to 0.217 m s^−1^, approximately 44% (Figure 10). Each configuration used a separately trained policy; Section 4.2 discusses this speed–stability trade-off and its implications for interpreting the support mechanism.

**Figure 11:**
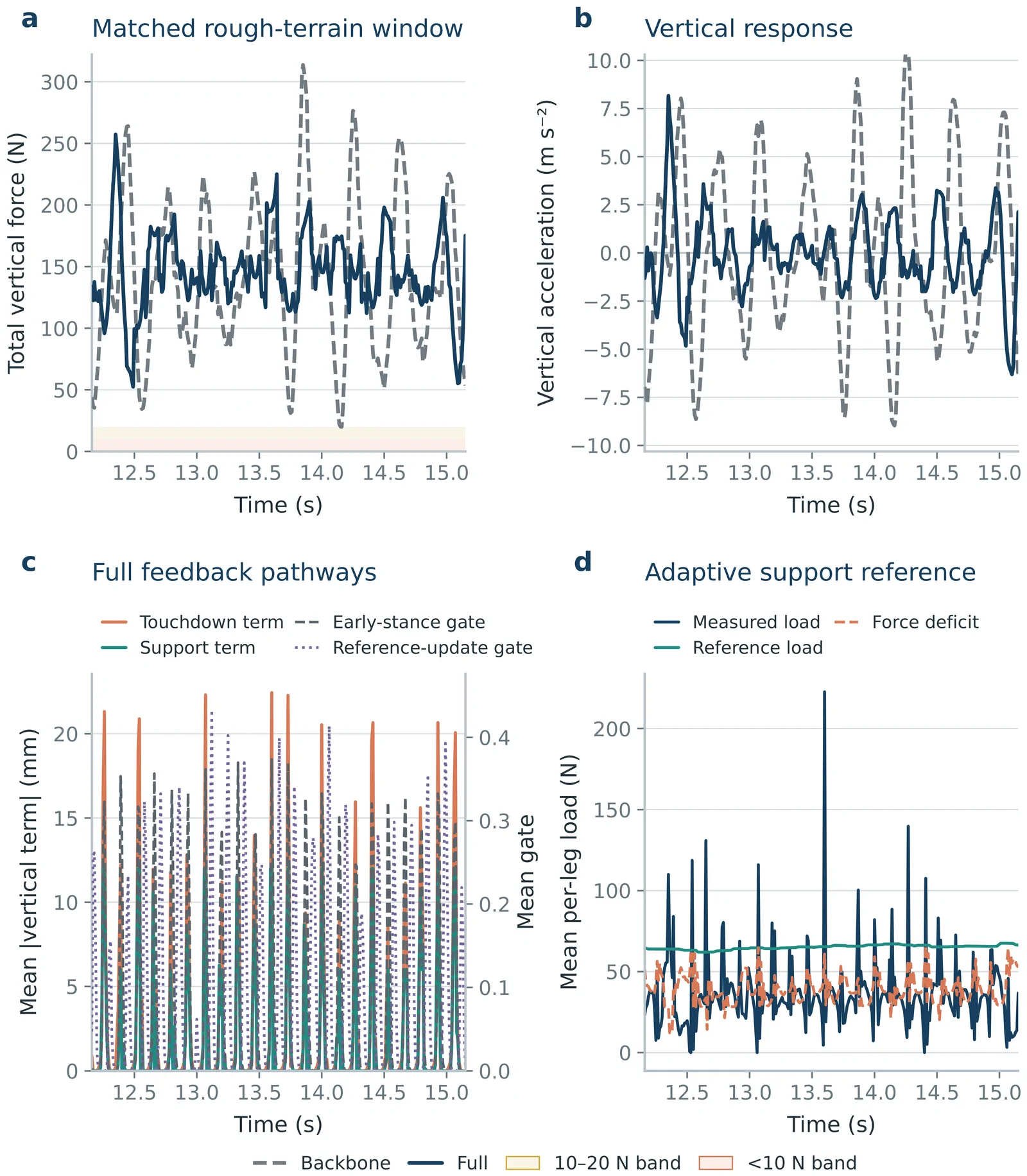
Representative support stabilization on the ±6 cm random rough terrain. Backbone and Full configurations receive the same 0.8 m s^−1^ command. (**a**) Total vertical contact force after 80 ms display smoothing; shaded intervals mark raw total force below 20 and 10 N. (**b**) Smoothed body vertical acceleration on shared axes. (**c**) Full-configuration touchdown/support corrections and their phase gates. (**d**) Mean per-leg contact load, support reference, and positive force deficit. The displayed interval spans 12.16–15.15 s of the corresponding 20 s rollouts.

In the separate representative Backbone–Full rough-terrain rollouts, Full halved total-force exposure below 20 N (6.35% to 3.10%) and reduced vertical-acceleration RMS from 5.64 to 4.75 m s^−2^. Figure 11 shows the associated touchdown and support activity.

## 4. Discussion

### 4.1. Relation to Privileged Learning and Rhythmic Control

This framework connects terrain-aware learning to terrain-height-blind execution through a shared rhythmic action representation. Teacher and student specify the same amplitude, cadence, and foot-placement variables, while the GSC-CPG converts them into contact-responsive trajectories. The student can therefore learn from teacher actions without changing the downstream control structure at deployment. This is the central role of the shared interface in the proposed method.

The design complements approaches that infer missing environmental information from proprioception. Rapid motor adaptation (RMA) uses an adaptation module to infer latent environmental information from recent proprioceptive history [27]. DreamWaQ jointly learns a proprioceptive policy and a context-aided state estimator with a privileged critic, without a separate teacher–student stage; its policy specifies joint-angle targets [28]. Here, a frozen teacher supplies both offline BC targets and scheduled online supervision, and the recurrent student modulates the rhythmic controller rather than specifying joint targets directly.

CPG-RL established reinforcement learning of oscillator modulation [17]. The present framework couples that type of structured control with privileged supervision and bounded phase and contact corrections shared by teacher and student. Its separation of descending commands, rhythm, interlimb coordination, and sensory feedback follows the biological organization that motivates the architecture [2,5,12]. The explicit control variables aid interpretation, although they also restrict the policy to the chosen rhythmic and foot-trajectory parameterization. RMA, DreamWaQ, and CPG-RL include hardware demonstrations; the present contribution is evaluated in simulation. The comparison here concerns information use and control structure, rather than a performance ranking across different platforms and test conditions.

### 4.2. Interpretation and Performance Trade-Offs

The matched experiment shows that teacher guidance remained useful after behavior cloning. Starting from the same BC model, guided students had lower failure rates, tracking error, body tilt, and action saturation than students trained with guidance disabled. The direction of these differences was consistent across the three paired training runs (Table 5 and Figure 4). Thus, the teacher contributed during subsequent policy optimization, rather than serving only to initialize the student. This finding applies to the tested 250-update joint-training stage and its low actor learning rate. Longer training or a different PPO schedule may change the relative value of continued supervision.

The action diagnostics reveal an important distinction. Saturation measures how often policy outputs exceed the execution range, whereas action-change RMS measures their variation between updates. Guidance improved the former but increased the latter. Better locomotion and fewer saturated outputs therefore came with greater temporal variation in the commands, rather than a uniform improvement in every aspect of control. Neither diagnostic alone establishes a change in actuator effort or wear.

The component tests provide a complementary view of the controller. The prescribed-input test shows that phase corrections follow changes in sensory timing and relax toward the nominal gait after withdrawal. The support comparison shows lower tilt and fall rate for Full, together with an approximately 44% reduction in speed. Because Support and Full use different trained policies and operate at different speeds, their performance difference cannot be assigned solely to support feedback. A fixed-policy, speed-matched comparison would be needed to separate these contributions. The current tests characterize the available phase and support responses without establishing a complete causal explanation of whole-robot robustness.

### 4.3. Operating Range and Simulation Limitations

The demonstrated operating range is straight-line locomotion on the tested rigid rough terrains and uphill slope. Transfer from ±6 cm to ±8 cm roughness extends the tested height range within a related geometric terrain family. Movable debris, yielding ground, granular sand, and deformable mud are not represented by the fixed triangle mesh. Dedicated clearance tests for isolated obstacles, including small obstacles that could be stepped over, were not performed.

Terrain-height blindness concerns the absence of terrain-height input, not the absence of state or contact information. Observation disturbances were applied to the actor, while the downstream controller retained direct simulator feedback. These tests therefore cover actor-observation stress under that feedback arrangement. Physical deployment must also account for errors in sensing, state estimation, and low-level feedback.

Statistical replication is limited to three student training seeds with a shared frozen teacher and BC dataset. The paired results and uncertainty intervals describe this training design; variability across independently trained teachers remains untested. Similarly, the absence of physical falls during the sampled slope exposure is an empirical outcome, not a zero-failure-probability or safety guarantee.

### 4.4. Timing, Computation, and Deployment Requirements

The implementation separates the 10 ms actor period from the 1 ms physics and controller updates. Joint feedback is refreshed at every physics step, whereas root, rigid-body, and contact measurements are held between actor updates. Consequently, the GSC-CPG integrates faster than most of its measurements are refreshed. Contact-dependent corrections use the latest available measurement during each hold interval.

This timing arrangement is relevant when considering numerical effects. A time-step sensitivity test would need to keep the actor and controller periods, measurement holds, and time-scaled quantities fixed to distinguish physical discretization from a change in the controller itself. Such a test was not performed here, so numerical convergence and the magnitude of any time-step effect remain unquantified. The reported results apply to the specified integration and measurement-update settings.

At deployment, the actor is evaluated at 100 Hz and the fixed four-leg controller at 1 kHz; teacher and critic computation is removed. The actor mean network contains 175,372 parameters, with 174,080 multiply–accumulates in its dense and GRU matrix products per robot per actor step. These analytical counts describe the network workload. They exclude nonlinearities, preprocessing, the GSC-CPG, inverse kinematics, joint control, state estimation, and communication, and therefore are not an end-to-end latency measurement.

The present study evaluates the framework in simulation. Physical-robot validation with onboard state and contact estimation is an important next step toward assessing its performance under real operating conditions.

## 5. Conclusions

We presented a framework that combines privileged teacher–student learning with a shared GSC-CPG for terrain-height-blind quadrupedal locomotion. Using the same rhythmic action representation for teacher and student allows terrain-aware action supervision during learning without supplying terrain-height observations at execution. In the matched simulations, continued guidance after BC initialization improved locomotion and reduced action saturation, although action variation increased. The guided policies also transferred to the tested held-out roughness and completed the unseen-slope exposure without physical falls.

The phase and support tests made the controller’s responses explicit, including reversible phase adjustments and a speed–stability trade-off in the support comparison. Together, these results support the use of a shared rhythmic interface for privileged locomotion learning within the tested simulation conditions. Broader terrain evaluation, independent teacher-training repetitions, and hardware validation with estimated state and contact feedback are needed to assess its wider applicability.

## Figures and Tables

**Table 1:** Principal ranges and bounded authorities of the shared gait-structured interface.

Component	Implemented Specification
Structured action	Environment/execution clips ±100/±4; amplitude range [1, 4], requested-cadence range [8, 30] rad s^−1^, and policy sagittal range ±0.04 m.
Cadence allocation	Common-owner time constant 0.75 s; per-leg cadence residual ±4 rad s^−1^; final per-leg command clipped to [8, 30] rad s^−1^.
Modal allocation	Nominal bounds $\left[-1.0,\;0.60\right]\times {\left[-0.20,\;0.20\right]}^{2}$ rad and total bounds $\left[-1.0,\;0.60\right]\times {\left[-0.60,\;0.60\right]}^{2}$ rad; persistent-source and nominal-state time constants 0.5/1.0 s.
Modal dynamics	Common gain 4; fast gains diag(3, 3, 2); decay diag(8, 8, 6) s^−1^.
Applied phase correction	Sensor-consistency, touchdown, and total per-leg limits 0.12/0.06/0.18 rad.
Vertical and yaw authority	Ground-reference range ±0.04 m; touchdown, late-search, and support limits 0.02/0.03/0.024 m; yaw-modal scalar limit 0.05 m (at most ±0.025 m per leg).

**Table 2:** Task-reward terms shared by the teacher and online student PPO policies. Here, ${\left(x\right)}_{+}=\max\left(x,0\right)$.

Term	Definition $\rho_{m,t}$	Weight $w_{m}$
Planar tracking	$\exp[-∥c_{xy}-v_{xy}{∥}_{2}^{2}/0.25]$	1
Tilt proxy	$∥g_{xy}^{\mathrm{proj}}{∥}_{2}^{2}$	−2
Mechanical power	$\sum_{j}\left|\tau_{j}{\dot{q}}_{j}\right|$	$-2\times 10^{-4}$
Excess contact force	$\sum_{i}(∥F_{i}{{∥}_{2}-180)}_{+}$	$-10^{-3}$
Action rate	$∥a_{t}^{100}-a_{t-1}^{100}{∥}_{2}^{2}$	$-10^{-2}$

**Table 3:** Training methods. Every deployed actor uses 68-D inputs; every online PPO method uses the same clean 128-D privileged critic. No-teacher methods start from random initialization; guided methods start from paired behavior-cloning (BC) checkpoints. Their optimization settings and training budgets are specified in the text. FF, feed-forward; MLP, multilayer perceptron; IID, independent and identically distributed.

Method	Actor	Teacher Guidance	Actor Observation
BC recurrent	GRU	Offline teacher targets	Clean
No-teacher FF	MLP	None	IID-corrupted
No-teacher GRU	GRU	None	IID-corrupted
Teacher-guided GRU (clean)	GRU	Scheduled action loss	Clean
Teacher-guided GRU (corruption training)	GRU	Scheduled action loss	IID-corrupted

**Table 4:** Analog actor-observation disturbances. Noise and episode bias are zero-mean Gaussian. Persistent noise standard deviation (SD) is its stationary value. Drift increments have SD equal to the listed coefficient multiplied by $\sqrt{\Delta t}$, with Δt=0.01 s; drift is clipped symmetrically at the listed limit. All magnitudes use the signal’s physical unit; drift coefficients use that unit per $\sqrt{s}$.

Signal (Unit)	IID Noise SD	Persistent Noise SD	Persistent Bias SD	Persistent Drift Coefficient	Persistent Drift Limit
Linear velocity (m s^−1^)	0.05	0.04	0.08	0.01	0.15
Angular velocity (rad s^−1^)	0.02	0.02	0.015	0.003	0.05
Projected gravity (dimensionless)	0.01	0.01	0.015	0.002	0.04
Joint-position error (rad)	0.005	0.004	0.004	0	0
Joint velocity (rad s^−1^)	0.10	0.12	0.05	0	0

**Table 5:** Effect of continued teacher guidance after BC initialization. Values pool two roughness levels and two actor-disturbance conditions; Δ is guided minus guidance off. Three BC-paired training seeds are crossed with three evaluation seeds and four fixed conditions (36 cells per method), with 128 environments and 2000 actor steps per cell. Endpoint-wise 95% intervals use 20,000 crossed paired resamples (Section 2.4). RMSE, root-mean-square error; RMS, root mean square.

Endpoint	Guided	GuidanceOff	Δ	95% Confidence Interval
Failure rate (events/robot-min)	1.097	1.479	−0.3815	[−0.5137, −0.2578]
Tracking RMSE (m s^−1^)	0.5272	0.5598	−0.0326	[−0.0531, −0.0189]
Tilt-proxy RMS	0.2021	0.2229	−0.0209	[−0.0321, −0.0133]
Mean forward velocity (m s^−1^)	0.5207	0.4887	0.0321	[0.0172, 0.0551]
Action saturation (fraction)	0.2301	0.4544	−0.2244	[−0.2466, −0.2058]
Raw action-change RMS	0.7920	0.7436	0.0484	[0.0325, 0.0606]
Execution-equivalent action-change RMS	0.7870	0.7381	0.0489	[0.0333, 0.0609]

**Table 6:** Five-method comparison under actor-observation stress. Entries give the mean and, in parentheses, sample standard deviation across three student training seeds. Each seed summary pools three evaluation seeds, two roughness amplitudes, and both disturbance conditions. These are complete training-procedure comparisons, not isolated teacher-loss effects. Cell-level RMS values are averaged as specified in Section 2.4.

Method	Failure Rate (Events/Robot-min)	Tracking RMSE (m s^−1^)	Tilt-Proxy RMS	Mean $v_{x}$ (m s^−1^)	Saturation (Fraction)
BC recurrent	1.393 (0.010)	0.541 (0.002)	0.205 (0.003)	0.491 (0.004)	0.274 (0.022)
No-teacher FF	2.706 (0.475)	0.573 (0.015)	0.259 (0.023)	0.469 (0.013)	0.534 (0.021)
No-teacher GRU	16.055 (7.865)	1.054 (0.137)	0.520 (0.136)	−0.076 (0.158)	1.000 (0.000)
Teacher-guided GRU (clean training)	1.054 (0.146)	0.517 (0.004)	0.195 (0.009)	0.521 (0.005)	0.225 (0.019)
Teacher-guided GRU (corruption training)	1.128 (0.061)	0.518 (0.001)	0.196 (0.006)	0.520 (0.002)	0.231 (0.019)

## Data Availability

The data presented in this study are available on request from the corresponding author.

## Supplementary Materials

The following supporting information can be downloaded at https://www.mdpi.com/article/10.3390/biomimetics11090677/s1, Video S1: Synchronized 12 s terrain-height-blind student rollout corresponding to Figure 8; Video S2: Representative 10 s GSC-CPG Backbone rollout on the ±6 cm random rough terrain; Video S3: Representative 10 s GSC-CPG Full-configuration rollout under the same condition; Video S4: Matched recurrent students trained with scheduled teacher guidance and with guidance disabled, corresponding to Figure 7 on the same ±6 cm random rough-terrain realization under clean actor observations and a $0.8\;m\;s^{-1}$ command; Video S5: Synchronized 20 s matched-policy replay on the unseen 5° uphill slope at the same command, using a body-yaw-following, world-up view with explicit heading cues and corresponding to panels (a–e) of Figure 5.

## Abbreviations

The following abbreviations are used in this manuscript:

BC	Behavior cloning
CPG	Central pattern generator
FF	Feed-forward
GAE	Generalized advantage estimation
GRU	Gated recurrent unit
GSC-CPG	Gait-structured sensor-consistency central pattern generator
IID	Independent and identically distributed
IK	Inverse kinematics
IMU	Inertial measurement unit
KL	Kullback–Leibler
MLP	Multilayer perceptron
MSE	Mean squared error
PD	Proportional–derivative
PPO	Proximal policy optimization
RL	Reinforcement learning
RMA	Rapid motor adaptation
RMS	Root mean square
RMSE	Root-mean-square error
SD	Standard deviation
ZOH	Zero-order hold
